# Clinical Remarks upon Surgical Cases in the Buffalo General Hospital—Amputation of Thigh—Removal of Large Tumor

**Published:** 1863-12

**Authors:** J. F. Miner


ART. III.—*Clinical Remarks upon Surgical Cases in the Buffalo General Hospital—Amputation of Thigh—Removal of large Tumor.*—By J. F. Miner, M. D.

Nov. 7, 1863.

Gentlemen:—The first case which we propose to present for your observation, is one of scrofulous disease of the knee joint. Your attention is particularly called to the appearance and condition of deformity which justifies the operation we are about to make. Amputation of the thigh you are aware is one of the most severe and dangerous operations in surgery; though the dangers depend greatly upon the causes which have produced the necessity for operation, and also upon the point in the thigh, where amputation is to be made, together with many other conditions and circumstances which must be clearly apparent to all. The fatality has been great after severe injuries and gun-shot wounds, while much better results have been obtained when amputation has been made for removal of disease, as in the case before you. The risks have also been found to be greatly diminished in proportion as the point of amputation was removed from the body; removal at the lower or middle third being very much more succcessful than at the upper third or hip joint.

This young lady is twenty-four years old, of decidedly scrofulous constitution, but without evidence of tuberculosis of the lungs. She has suffered from this disease of the knee joint for a good many years, and attributes the first cause to injury received when a young girl by a fall. For the last five years she has suffered much more severely, and there has been constant purulent discharge from the various openings you now observe. The tissues around the knee are infiltrated and hardened, while the motions of the joint are lost, and the leg is flexed strongly upon the thigh. A probe passes deeply into the structures of the joint but does not detect denuded, carious or necrosed portions of bone, and yet there can be no doubt that the articulating surfaces of the joint are extensively diseased and ulcerated. This drain upon the system, together with the pain, which is constant and severe, will be observed to have reduced the general strength and produced a condition of anæmia which has been rapidly increasing for the past few months. This increasing prostration indicates that nature is unable to bear up against so extensive and continued disease, and that removal of the leg and knee is a necessity, for the long continuance of life.

Nothing but this necessity could induce such operation, since it is so full of danger. Amputation of the thigh has proved fatal in so large a proportion of cases that it has been almost abandoned by surgeons, indeed when made after gun-shot and other injuries, it is so uniformly fatal as to be the rule, with only here and there an occasional exception. "Amputation of a limb is the last resource and opprobrium of surgery, as death is of the practice of physic; it being notwithstanding impossible to do impossibilities, and save a limb or a life which can no longer be preserved." The operation itself is very simple and easily performed; even a poor surgeon may make it very well.

There are a great many questions connected with this subject, which however it is no part of my purpose to discuss; they have been under consideration by the profession for hundreds of years. and yet the New York Academy of Medicine, propose at the next meeting, a discussion upon amputations in gun-shot fractures of the thigh; showing that the principles of practice are not even now fully settled. After this discussion we have no doubt differences of opinion will be entertained upon many points connected with amputations of the thigh, both after injury and on account of disease.

The diseased mass which is here removed has some interest to you, and for the purpose of showing the condition of the structures of the joint, the bones and articulating cartilages, I will lay it open before you. The patella is denuded of cartilage in nearly its whole under and articulating surface. The cartilages of the joint are all thickened and ulcerated, while the heads of the bones are also carious, or in condition of disease which answers to ulceration in the soft parts.

It is manifest upon examination, that this condition of extensive disease could never be recovered from, and that the only possible chance of preserving life was in making the amputation. It has been made with a loss of not more than two or three ounces of blood, a less quantity than I have ever seen in making amputation of the thigh. This was due mainly to the perfect compression of the arteries by the tourniquet and its not being tightened upon the veins, allowing free return of the circulation, until the very moment of making the incision, together with the almost instantaneous ligation of the larger vessels. The small loss of blood is in this case of great importance, for our patient has no blood to spare. The full effects of sulphuric ether has also prevented, I believe, in a great degree, the shock which is usual in making capital operations. We are yet exposed to many dangers, but have done what we deem best, and must abide the results.

*The 2d case* which we have for operation this morning is a very large tumor, situated upon the side of the head and neck, but in such situation that it can be quite safely removed. It has attained great size, appearing almost like a second head upon the side of the natural or original one. It appears in the location where encysted tumors of various kinds often make their appearance. It is probably what is called fatty or steartomatous tumor, but it is hard and unyielding for such growth, and this throws some doubt upon the case, indeed there is apt to be doubt in all these cases, and one can hardly be positive without exploration either previous to, or after operation. The question of most vital importance and the only one which would greatly influence our practice, may be answered with almost positive certainty. That it is benign, and not of malignant character is made probable, certain I may say, by 1st, its history. It has been present in this form for several years; has not increased in size or been painful; has not affected the general health; 2d, by its appearance and location. It is lobulated, but not hard enough for scirrhus; it is not cerebriform or encephaloid cancer, because such disease is much more rapid in its course and termination. Though malignant disease may involve any, and all the structures of the system, still it is not common for it to commence in this location and present this appearance, or attain to this size.

It is worthy attention, since clinical observation will soon enable you to determine with tolerable certainty between the benign and malignant growths; though it must be confessed that the instances are not rare, where deep seated tumors will evade all distinction, their true characters cannot be determined.

The tumor has been removed with the distinct cyst, which has probably been formed by the cellular tissue being gradually condensed by the immense growth of the tumor. Upon section it is seen to be fatty, and the hardness and unyielding quality of it, is caused by the hypertrophy of the cellular tissue, of which the mass is partially composed, which seems almost like fibrous degeneration.

This old gentleman is about 70 years of age, and shows considerable resolution, is desiring the removal of this tumor; he is, however, vigorous and active, and will probably bear the operation very well.

We have in the wards several other patients waiting impatiently for operation, which we must postpone until another time, while it will afford us pleasure to present before you each week, such cases as seem of interest. The number may not be great, but they possess additional interest in being as politicians sometimes say, "fresh from the people," that is, they are such as every one who practices surgery will be called upon to treat.

The Buffalo Genaral Hospital now offers those who require it, *gratuitous medical and surgical treatment*, and thus will perhaps make its advantages for clinical observation equal, or even superior to any other institution west of New York and Philadelphia. As one of the attending surgeons, I most cordially invite you to its advantages, happy if I may in the least contribute to your surgical opportunity.

